# Combinational Effect of* Rumex tingitanus* (Polygonaceae) Hexane Extract and* Bacillus thuringiensis*  *δ*-Endotoxin against* Spodoptera littoralis* (Lepidoptera: Noctuidae)

**DOI:** 10.1155/2018/3895834

**Published:** 2018-08-08

**Authors:** Dhekra Mhalla, Dalel Ben Farhat-Touzri, Slim Tounsi, Mohamed Trigui

**Affiliations:** ^1^Laboratory of Biopesticides, Center of Biotechnology of Sfax, University of Sfax, P.O. Box 1177, 3018 Sfax, Tunisia; ^2^Research Unit “Coastal and Urban Environments” University of Sfax, Sfax Preparatory Engineering Institute, BP 1172, 3018 Sfax, Tunisia

## Abstract

The increasing insect resistance against* Bacillus thuringiensis *delta-endotoxins is a serious problem which makes it urgent to look for new eco-friendly strategies. Combining these toxins with other biomolecules is one of the promising strategies against insect pests. In this work, we evaluated the bioinsecticidal potential of* Rumex tingitanus* extracts and* B. thuringiensis* strain BLB250 against* Spodoptera littoralis *(Lepidoptera: Noctuidae) larvae. The chemical composition of the hexane extract, the most active fraction, was analyzed to validate the correlation between chemical composition and biological activity. Among the tested extracts, only the hexanic extract showed toxicity against first and second instar larvae with LC_50_ of 2.56 and 2.95 mg g^−1^, respectively. The* Bacillus thuringiensis *BLB250 delta-endotoxins showed toxicity with an LC_50_ of 56.3 *μ*g g^−1^. Therefore, the investigated combinational effect of BLB250 delta-endotoxins and* R. tingitanus* hexane extract proved significant synergistic effect against* S. littoralis* larvae. The GC-MS analysis of* R. tingitanus *hexane extract showed the richness of this extract in phytosterols such as *β* and γ-sitosterol (48.91%), campesterol (6.43%), and *β*-amyrin (8.92%) which are known for their insecticidal activity. This novel finding highlights the potential use of this combination against insect pests to prevent the appearance of resistance problems.

## 1. Introduction

*Spodoptera littoralis* (Boisduval) (Lepidoptera: Noctuidae) is one of the most serious pests which are known for their high polyphagia, voracity, rapid development, and worldwide spread. It can attack 87 species of economically important plants including cotton, tomatoes, legumes, cruciferous, artichokes, strawberries, forage, corn, peppers, and other crops in Africa, Asia, and Europe [1, 2]. Synthetic insecticides are extensively used to control this pest worldwide. However, their indiscriminate application has led to the development of insect resistance, environmental pollution, residual toxicity, and serious threats to the nontarget organisms including predators, pollinators, fish, and human beings [3]. According to the current European guidelines for agri- and horticulture, a combination of botanical pesticides and beneficial organisms is one of the potentially desirable alternatives in the integrated pest management. Plant extracts or metabolites and microbial formulations provide an interesting alternative to the conventional chemical insecticides. These biopesticides may be a promising part of insect biocontrol technology in the future [4].* Bacillus thuringiensis *endotoxins (BLB250 endotoxins) are the most widely used microbial insecticides in the world [5]. They are toxic against different species of insect pests such as lepidoptera, diptera, and coleoptera. Many preparations based on* B. thuringiensis* have significant biochemical and physiological effects on* S. littoralis* larvae. However, developing resistance to this entomopathogenic bacterium requires finding out new methods for pest control [6]. On the other hand, more than 2000 plant species are known for their insecticidal activities because they are a rich source of secondary bioactive metabolites [7]. Plant extracts or metabolites are considered one of the most important control methods which are less hazardous to nontarget organisms, environmentally safe, and biodegradable [4]. The insecticidal potential of various plants against* S. littoralis* has been demonstrated by many researchers [4, 8, 9]. In this work, we evaluated the bioinsecticidal potential of* Rumex tingitanus *extracts and* B. thuringiensis* strain BLB250 against* S. littoralis* larvae. The chemical composition of the hexane extract, which is the most active fraction, was analyzed to prove the correlation between chemical composition and biological activity.

## 2. Materials and Methods

### 2.1. Plant Material

*R. tingitanus* leaves were harvested in March of 2015 from Sfax Tunisia (latitude 34°46'31” N and longitude 10°45'59” E). The leaves were dried and stored in the Laboratory of Biopesticides and a voucher specimen was deposited in the Center of Biotechnology of Sfax, Tunisia.

### 2.2. Bacterial Strain

*B. thuringiensis* strain BLB250 used in this study was isolated in the Laboratory of Biopesticides from soil. The biochemical and molecular characterization as well as the produced delta-endotoxins have been previously studied by Benfarhat-Touzri et al. (2016) [10].

### 2.3. Insect

*S. littoralis *larvae used in these bioassays were reared on an artificial diet in the Laboratory of Biopesticides and maintained under standard conditions at 23°C, 65% relative humidity, and 16:8 h light:dark photoperiod. They fed on an artificial semisolid diet, consisting of a mixture of wheat germ, beer yeast, maize semolina, nipagine, ascorbic acid, wessan salt, sorbic acid, benzoic acid, agar, and water [11]. The diet was poured into sterile Petri dishes, allowed to cool thoroughly, and then stored at 4°C for up to 7 days. The adults were fed by a 10% sucrose solution.

### 2.4. Preparation of Plant Extracts

Fresh leaves of* Rumex tingitanus *were dried and fine powdered. The obtained powder (1 kg) was macerated with aqueous ethanol (4 L of ethanol/water, 4:1, v/v) by occasional shaking at room temperature. After 48 h, the extract was filtered and concentrated under vacuum. The resulting hydroalcoholic extract (RtEtOH-H_2_O; 203 g) was solubilized in water and fractionated with n-hexane and then by ethyl acetate to obtain a n-hexane fraction (RtHexF), ethyl acetate fraction (RtEtOAcF), and water fraction (RtWF), respectively [12].

### 2.5. GC-MS Analysis of* R. tingitanus *Hexane Extract

The quantitative analysis of* R. tingitanus* hexane extract was established by GC-MSHP model 6980 inert MSD (Agilent Technologies, USA), equipped with a mass selective detector (MSD5973, ionization voltage 70 eV; Agilent, Santa Clara, CA) and capillary column HP-5MS (30 m length, 0.25 mm diameter, and 0.25 mm film thickness). Helium was used as a carrier gas at a flow rate of 1 ml min^−1^. The sample was injected with a split mode 1/100. The temperature of the injector was maintained at 280°C. The original oven temperature was set to 50°C for 2 min and ramped to 300°C at a rate of 5°C min^−1^ and then this temperature was held for 8 min. The components were identified by careful examination of fragmentation patterns and spectral data obtained from the Wiley Registry of Mass Spectral Data 7th edition (Agilent Technologies, Inc.) and the National Institute of Standards and Technology 05 MS (NIST) library data. This determination was carried out in duplicate.

### 2.6. Preparation of* B. thuringiensis* BLB250 Delta-Endotoxins

*B. thuringiensis* strain BLB250 was grown on T3 solid culture medium for 72 h at 30°C [10]. The spore-crystal mixture collected in 1 M NaCl cold solution was harvested by centrifugation for 10 min at 10,000 × g and washed twice with cold distilled water. 50 *μ*L of spore-crystal mixtures were further solubilized in 50 mM NaOH and incubated during 2 h at 30°C [13]. BLB250 delta-endotoxin concentrations were determined by Bradford method [14].

### 2.7. Bioinsecticidal Assays

#### 2.7.1. Chronic Toxicity

The chronic toxicity of* R. tingitanus* extracts (RtEtOH-H_2_O, RtEtOAcF, RtHexF, and RtWF) and* B. thuringiensis* delta-endotoxins against* S. littoralis* larvae were examined according to the protocol described by Benfarhat-Touzri et al. [13], with slight modifications. Five concentrations of each tested extract (0.3125-5 mg g^−1^) and BLB250 delta-endotoxin (10-100 *μ*g g^−1^) were prepared separately to determine the fifty and ninety percent lethal concentrations (LC_50_ and LC_90_) values. One gram of artificial diet was placed in a sterile Petri dish and blended with 100 *μ*L of the appropriate concentration of extracts and delta-endotoxin. After complete drying, ten insects of the newly molted first, second, third, and fourth instar larvae of* S. littoralis* were added to the mixture. Then, the plates were incubated for 48 hours under standard conditions at 23°C, 65% relative humidity, and 16:8 h light:dark photoperiod. The untreated control groups were prepared in the same experimental conditions but the diet was impregnated with 50% of ethanol or buffer solution free of BLB250 delta-endotoxin. Each treatment was performed in triplicate. The larval mortality was determined within 48 hours. LC_50_ and LC_90_ values were calculated by probit analysis.

#### 2.7.2. Growth Inhibition

In order to assess the efficiency of* R. tingitanus* on larval growth, the artificial diet containing hexane extract in 5 concentrations (0.3125, 0.625, 1.25, 2.5, and 5 mg g^−1^) was used. The plates were prepared as described above. Then, the newly molted fourth instar larvae of* S. littoralis *were placed individually in sterile Petri dishes. After incubation in the growth room (16:8 h light:dark, 23°C), the larvae were weighed after 48 hours. The result was expressed as growth inhibition percentage (%) using the following formula:

(1) $$\begin{array}{c} \text{Growth inhibition (\%)}=100-\left[\;\left(\;\frac{T}{C}\;\right)\times 100\;\right] \end{array}$$

T is the sample larval weight and C is the control larval weight. Ten larvae were tested for each concentration. Three repetitions were performed for each treatment [15].

#### 2.7.3. Joint Effect Studies

The* R. tingitanus* hexane extract (RtHexF) at concentrations of 0.3125, 0.625, 1.25, 2.5, and 5 mg g^−1^ and BLB250 delta-endotoxins (30 *μ*g g^−1^) were blended together in binary combinations. For each tested mixture, three repetitions were performed. The toxicity experiments were carried out as described for chronic toxicity where larval mortalities were determined after 48 hours and the actual mortalities were compared to the expected mortalities based on the following formula:

(2) $$\begin{array}{c} E=O_{a}+O_{b}\left(\;1-\frac{O_{a}}{100}\;\right) \end{array}$$

E is expected mortality and O_a_ is the observed mortality of BLB250 and O_b_ is the observed mortality of RtHexF at the given concentration.

The Chi-squared test *χ*^2^ was used to designate the effects of each mixture which can be additive, antagonistic, or synergistic.

(3) $$\begin{array}{c} \chi^{2}=\frac{{\left(O_{m}-E\right)}^{2}}{E} \end{array}$$

E is the expected mortality from the binary combination and O_m_ is the observed mortality; *χ*^2^ with df = 1 and p = 0.05 is 3.84.

This test differentiates the results into three categories. A pair with *χ*^2^ values < 3.84 indicates an additive effect, with *χ*^2^ values > 3.84 and O_m_ > E indicates a synergistic effect, whereas a pair with *χ*^2^ values > 3.84 and O_m_ < E indicates an antagonistic effect [8].

## 3. Results

### 3.1. Chemical Analysis of* R. tingitanus *Hexane Extract

Analysis of RtHexF by GC-MS method revealed a complex mixture of chemical families consisting essentially of phytosterol (67.48%) and fatty acid compounds (3.6%) (Table 1). Four major compounds (>6%) were identified as *γ*-sitosterol (26.32%), *β*-sitosterol (22.59%), *β*-amyrin (8.92%), and campesterol (6.43%) followed by fatty acids (palmitic and linolenic acids), stigmasta-3,5-dien-7-one (1.49%), *δ*-5-Ergosterol (0.52%), and Camphor (0.68%).

### 3.2. Insecticidal Potential of* R. tingitanus *against* S. littoralis*

#### 3.2.1. Toxicity

Chronic toxicity of* R. tingitanus *extracts, measured as mortality within 48 hours, was determined by oral application to 1st, 2nd, 3rd, and 4th instar larvae of* S. littoralis. *The results displayed in Table 2 indicated that among the tested extracts, only the hexane extract (RtHexF) exhibited remarkable larvicidal activity. The larval mortalities were increased with the rise of the extract concentration. After a forty-eight-hour treatment, the highest concentration (5 mg g^−1^) caused 70 ± 0.8%, 60 ± 2.4%, and 10 ± 0.0% of mortality against 1st, 2nd, and 3rd instar larvae of* S. littoralis*, respectively. However, the exposure of larvae to the other three extracts did not cause any mortality. No mortality was observed against 4th instar larvae, either. No toxicity was produced from the negative control over the 48 h.

Based on the comparison of the lethal concentrations, the chronic toxicity of RtHexF against 1st instar larvae was slightly higher (LC_50_ = 2.56 mg g^−1^; LC_90_ = 6.02 mg g^−1^) than the toxicity against 2nd instar larvae (LC_50_ = 2.95 mg g^−1^; LC_90_ = 6.65 mg g^−1^). For the 3rd instar larvae, the lethal concentrations could not be determined because the highest applied concentration caused a lower mortality rate than 50% (Table 3).

#### 3.2.2. Growth Inhibition

**Figure 1:**
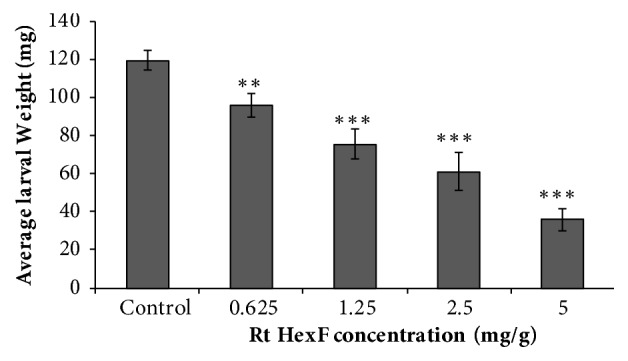
Growth inhibitory action of* R. tingitanus *hexane extract on* Spodoptera littoralis* after 48 hours of feeding on a treated diet. Ten fourth instar larvae of* S. littoralis *were exposed to different concentrations of Rt hexane extract and larvae mass was recorded at 48 h. Each treatment was replicated three times. Error bars represent the standard deviation of three replications. Superscripts *∗∗* and *∗∗∗* denote statically significant at* P* < 0.01,* P* < 0.001 with comparison to control.

A significant growth inhibition was observed in 4th instar larvae of* S. littoralis *after the oral application of* R. tingitanus* hexane extract (Figure 1). 70% inhibition rate was obtained after exposing of 5 mg g^−1^ hexane extract.

### 3.3. Insecticidal Potential of* B. thuringiensis *BLB250 against* S. littoralis*

The chronic toxicity of BLB250 delta-endotoxins was investigated by oral application to second instar* S. littoralis *larvae. The BLB250 delta-endotoxins exhibited a high insecticidal activity with LC_50_ of 56.3 ± 5.83 *μ*g g^−1^. No activity was determined for the control over the test period (Table 3).

### 3.4. Synergistic Effect of* R. tingitanus* Hexane Extract and* B. thuringiensis* Delta-Endotoxin

In order to improve toxicity and decrease the* S. littoralis* resistance to* B. thuringiensis *toxins, we have combined BLB250 delta-endotoxins (30 *μ*g g^−1^) with* R. tingitanus* hexane extract at concentrations of 0.3125, 0.625, 1.25, 2.5, and 5 mg g^−1^ against 1st instar larvae and 0.625, 1.25, 2.5, and 5 mg g^−1^ against 2nd instar larvae. In total, 9 binary combinations were tested against 1st and 2nd instar larvae of* S. littoralis*. As shown in Table 4, the results analysis indicated that an addition of a fixed delta-endotoxin concentration to the increasing concentrations of hexane extract improved the lethal effect. All the tested binary mixtures showed synergistic effects either against 1st and 2nd instar larvae of* S. littoralis* except that of the lowest concentrations. The highest *χ*^2^ value (10.9) was recorded for the BLB250 and RtHexF combination at 5 mg g^−1^.

## 4. Discussion

Chemical insecticides are extensively used against a broad range of bioaggressors such as insects, fungi, bacteria, and viruses. However, their adverse toxicological effects and the resistance developed by pests have urged a continuous search for safer methods [4]. Currently, the plant extracts are considered as a potential source of bioactive compounds which are potentially useful against diverse groups of insect pests [8].

The present study emphasized, for the first time, the promising insecticidal activities of* R. tingitanus* hexane extract against lepidopteran larvae. The mode of action of this insecticide varies according to the larval stage. It is a larvicide against 1st and 2nd instar larvae (LC_50_ = 2.56 mg g^−1^; LC_50_ = 2.95 mg g^−1^, respectively) and an antifeedant against 4th instar larvae. The larvacidal properties of* R. tingitanus* hexane extract to* S. littoralis* could be attributed to the presence of high percentage of compounds which are known for their potential insecticidal effect, particularly *β* and *γ*-Sitosterol and *β*-amyrin. In fact, Saeidnia et al. [16] reported the larvicidal activity of *β*-sitosterol. It was demonstrated that *β*-Sitosterol showed insecticidal, antifeeding, and insect growth regulatory activities against* Spodoptera littoralis* larvae [17]. Kannan et al. [18] showed that *β*-amyrin has antifeedant and growth regulating activities against* Spodoptera litura* (Lepidoptera: Noctuidae). In addition, minor compounds of the RtHexF, such as Stigmasta-3,5-dien-7-one and camphor, have also reported for their potential insecticidal activity [19, 20]. The tests carried out on the* Spodoptera frugiperda* larvae revealed the potential insecticidal activity of* Castela coccinea* including Stigmasta-3,5-dien-7-one [21]. Pavela et al. [8] proved the effectiveness of camphor against* S. littoralis. *Therefore, the diversity of major and minor compounds presents in the plant extract and the synergy between them should be taken into account for their insecticidal activity. From these results, the* R. tingitanus* hexane extract could be used for the biological control of* S. littoralis* larvae. Furthermore, lepidopteran pests control using* B. thuringiensis* toxins is currently preferred because they exhibit no threat to the environment and human health [22]. In this work, we have demonstrated the effectiveness of* B. thuringiensis* BLB250 delta-endotoxins against* S. littoralis *with impressive LC_50_ of 56.3 ± 5.83 *μ*g g^−1^. In order to improve the lethal effect and prevent the development of certain resistance cases, the combinational effect between* R. tingitanus* hexane extract and* B. thuringiensis *delta-endotoxins was investigated. The analysis of the results revealed the dominance of synergistic effects of mixing these two natural biomolecules, against 1st as well as 2nd instar larvae of* S. littoralis. *This combination may cause an as high as 100% efficacy in terms of toxicity for lepidopteran larvae compared to the control. Thus, our findings in this context may be considered as an outstanding way of biological pest management. The high cost of microbial toxin productions could be considerably lowered by using lower quantities of bacterial toxin mixed with plant extracts which approximately doubles the larvicidal effect. In fact, adding a lower amount of BLB250 delta-endotoxins (30 *μ*g g^−1^ <LC_50_) improved the toxicity of* R. tingitanus* hexane extract and vice versa. However, the assessment of the mode of action of the combinational effect of BLB250 delta-endotoxins and the plant extract against* S. littoralis* larvae may be a complex process which deserves further investigation. The synergism could be explained by inhibiting the* S. littoralis* larvae capacity to use detoxifying enzymes and the disruption of the integrity of the gut due to delta-endotoxins fixation on the midgut epithelial cell at specific receptors leading to the insect starvation and death [4, 23]. Therefore, the combinational effect or synergism between plant extracts and microbial or insecticide control agents cause more aggressive and a longer-lasting effects. The identification of these botanical components within mixtures has the potential to develop more effective and more economical biopesticides [24]. In fact, this joint-action may be an ideal solution to prevent the appearance of certain resistance cases caused by the systemic and repeated application of synthetic insecticide or* B. thuringiensis* delta-endotoxins [4].

## 5. Conclusion

In summary, it can be noted that compounds contained in* R. tingitanus* hexane extract could be a new alternative to chemical insecticides and could be used in the development of new natural insecticides. Mixing this extract with* B. thuringiensis *delta-endotoxins might be an ideal solution to delay or attenuate the insect resistance to* B. thuringiensis *toxins. These combinations may be integrated into an insecticidal formulation active against some Lepidoptera larvae. However, further tests need to be performed to investigate the mode of action and cost-efficacy of these combinations in greenhouses and fields.

## Figures and Tables

**Table 1:** Chemical composition of hexane extract prepared from *R. tingitanus *leaves.

**Compound**	**Rt (min)** ^**a**^	**KI** ^**b**^	**Peak area (**%**)**^**c**^
Camphor	9,754	1146	0.68
6.10.14-Trimethyl-2-pentadecanone	26.429	1847	0.40
Palmitate Methyl	28.049	1928	0.15
Palmitate Ethyl	29.360	1996	1.96
9.12-Octadecadienoic acid Methyl	31.214	2093	0.14
9.12.15-Octadecatrienoic acid Ethyl	31.335	2113	0.25
Trans-phytol	31.606	2117	0.90
9.12-Octadecadienoic acid Methyl	32.412	2155	1.21
9.12.15-Octadecatrienoic acid Ethyl	32.540	2215	1.85
***Phytosterols***			
*δ*-5-Ergosterol	39.67	3029	0.56
Campesterol	42.359	3131	6.43
Stigmasterol	44.687	3170	1.17
*β* -Sitosterol	45.463	3187	22.59
*β*-amyrin	46.375	3337	8.92
*γ* -Sitosterol	47.068	3351	26.32
Stigmasta-3.5-dien-7-one	48.176	3432	1.49

∑ Identified compounds			75.02

^a^Rt: retention time.

^b^KI: Kovats indices on HP-5MS capillary column with reference to C_10_–C_22_ n-alkanes injected in the same conditions.

^c^%: percentages are the means of two runs and were obtained from electronic integration measurements using a selective mass detector.

**Table 2:** Chronic toxicity of *R. tingitanus *extracts against *S. littoralis* larvae within 48 h.

**Treatment**	**Concentration (mg g** ^**-1**^ **)**	**Mortality (**%**)**			
		**1st instar**	**2nd instar**	**3rd instar**	**4th instar**
**RtEtOH-H**_**2**_ **O**	**2.5**	0	0	0	0
	**5**	0	0	0	0

**RtEtOAcF**	**2.5**	0	0	0	0
	**5**	0	0	0	0

**RtHexF**	**0.3125**	10 ± 1.0	0	0	0
	**0.625**	23.3 ± 1.1	15 ± 0.2	0	0
	**1.25**	30 ± 0.5	25 ± 1.0	0	0
	**2.5**	50 ± 0.5	50 ± 1.0	0	0
	**5**	70 ± 0.8	60 ± 2.4	10 ± 0.0	0

**RtWF**	**2.5**	0	0	0	0
	**5**	0	0	0	0

**Negative control **	-	0	0	0	0

Average mortality in % obtained after application of *R. tingitanus* extracts: Rt EtOH-H_2_O; hydroalcoholic extract, RtHexF; n-hexane fraction, RtEtOAcF; ethyl acetate fraction and RtWF; water fraction mixed with 1 g of artificial semisolid diet cubes. Larval mortality against the 1st to 4th instar larvae was determined after 2 days.

**Table 3:** Lethal concentrations (LC_50_ and LC_90_) of *R. tingitanus *hexane extract (mg g^−1^) and *B. thuringiensis *delta-endotoxin BLB250 (*μ*g g^−1^) against *S. littoralis* larvae.

**Instar larvae**	**LC**_**50**_ ** (mg g** ^**-1**^ **)**	**LC**_**90**_ ** (mg g** ^**-1**^ **)**
***RtHexF***		
**1st instar**	2.56 ± 0.56	6.02 ± 1.41
**2nd instar**	2.95 ± 0.65	6.65 ± 1.73
***BLB250***	**LC**_**50**_ ** (**μ**g g**^**-1**^**)**	**LC**_**90**_ ** (**μ**g g**^**-1**^**)**
**2nd instar**	56.3 ± 5.83	-

**Table 4:** Joint effect analysis for *B. thuringiensis *BLB250 delta-endotoxins mixed with different concentrations of *R. tingitanus* hexane extract (RtHexF) against 1st and 2nd instar larvae of *S. littoralis* within 48 h.

**Instar larvae**	**Compound** **a**	**Compound** **b**	**Larval mortality (**%**)**					
			**Compounds**		**Binary mixtures**			
			**Observed a**	**Observed b**	**Observed**	**Expected**	**χ** ^2^	**Effect**
1st instar	*BLB250*	RtHexF (0.3125 mg g^−1^)	30	10	40	37	0.24	Additive
	*BLB250*	RtHexF (0.625 mg g^−1^)	30	23.3	60	46.31	4.05	Synergistic
	*BLB250*	RtHexF (1.25 mg g^−1^)	30	30	70	51	7.08	Synergistic
	*BLB250*	RtHexF (2.5 mg g^−1^)	30	50	90	65	9.62	Synergistic
	*BLB250*	RtHexF (5 mg g^−1^)	30	60	100	72	10.90	Synergistic

2nd instar	*BLB250*	RtHexF (0.625 mg g^−^1)	30	10	45	37	1.73	Additive
	*BLB250*	RtHexF (1.25 mg g^−1^)	30	15	60	40.5	9.40	Synergistic
	*BLB250*	RtHexF (2.5 mg g^−1^)	30	50	90	65	9.61	Synergistic
	*BLB250*	RtHexF (5 mg g^−1^)	30	60	100	72	10.88	Synergistic

## Data Availability

No data were used to support this study.
